# Multilayer pH-Responsive Hydrogels Fabricated via Two-Step Ionic Crosslinking: Towards Advanced Wound Dressing Materials

**DOI:** 10.3390/gels11100840

**Published:** 2025-10-21

**Authors:** Gianluca Ciarleglio, Virginia Clarizia, Elisa Toto, Maria Gabriella Santonicola

**Affiliations:** Department of Chemical Engineering Materials Environment, Sapienza University of Rome, Via del Castro, Laurenziano 7, 00161 Rome, Italy; gianluca.ciarleglio@uniroma1.it (G.C.); clarizia.1997800@studenti.uniroma1.it (V.C.); elisa.toto@uniroma1.it (E.T.)

**Keywords:** alginate hydrogel, multilayer, pH responsive, chitosan coating, burn care

## Abstract

The design of hydrogel-based materials for wound care management requires the integration of multiple functionalities, including the capacity to maintain hydration, to prevent infection, and to adapt to the dynamic wound microenvironment. In this study, we fabricated innovative pH-reactive multilayer hydrogel patches based on ionically crosslinked alginate and incorporated with bioactive compounds, including Manuka honey, hyaluronic acid, and *Ribes nigrum* extract. The multilayer structure is coated with chitosan to improve water affinity and pH response. The patches are designed to respond to variable pH conditions typical of wound environments, with potential applicability to burn wounds. The hydrogel materials are characterized in terms of water content, swelling behavior, and water vapor transmission rate (WVTR). The chitosan-coated multilayer hydrogel exhibited high water uptake (swelling ratio up to 22.11 ± 0.25; water content 95.48 ± 0.05%) and controlled WVTR (~3450–3850 g/m^2^·day^−1^), while degradation remained below 42% at pH 8 compared to >80% in single layers. Microstructural analysis is performed via optical microscopy to assess the morphology and uniformity of the multilayer system, while chemical characterization is conducted using Fourier-transform infrared (FTIR) spectroscopy. The results highlight the ability of the designed material to respond to pH variations and to accommodate bioactive agents within a structurally stable and hydrated network, suggesting its suitability for future investigations into controlled release applications.

## 1. Introduction

Wound care management is a critical aspect of clinical treatment aimed at promoting rapid tissue regeneration, preventing infection, and minimizing scar formation. In particular, burn injuries represent a significant global health challenge, accounting for over 11 million medical cases annually, many of which result in severe tissue damage, prolonged hospitalization, and substantial socioeconomic impact [1]. In this context, conventional dressing materials, such as cotton gauzes and films, primarily provide passive protection but often fail to maintain optimal moisture balance or respond to the dynamic physiological environment of the wound. These limitations have prompted growing interest in the engineering of advanced materials that can actively modulate the wound microenvironment while delivering therapeutic agents These materials are necessary for the effective healing of complex wounds requiring stringent control of infections, exudate levels, and local pH microenvironment [2,3].

Hydrogels, owing to their high water content, biocompatibility, and tunable mechanical properties, have emerged as promising materials for wound care applications [4]. Their soft, viscoelastic nature mimics native extracellular matrix, enabling protection from mechanical trauma while maintaining a moist environment able of enabling cellular migration and proliferation [5]. Moreover, hydrogel systems are particularly advantageous for incorporating bioactive compounds that can enhance healing through antibacterial, antioxidant, or regenerative effects [6,7,8].

Among naturally derived polymers used in hydrogel fabrication, sodium alginate, a linear polysaccharide extracted from brown algae, has gained attention due to its ability to form ionically crosslinked networks in the presence of divalent cations, such as Ca^2+^ [9,10]. The resulting “egg-box” structures provide mechanical integrity, conformability, and good swelling behavior [11]. Nevertheless, alginate-based hydrogels often require functional enhancement through the incorporation of therapeutic agents to achieve optimal performance in complex wound environments such as burns. For instance, Bergonzi et al. developed 3D-printed chitosan/alginate hydrogels loaded with 0.75% *w*/*v* silver sulfadiazine, demonstrating controlled drug release and effective antimicrobial activity against *Staphylococcus aureus* and *Pseudomonas aeruginosa*, highlighting their potential use in burn wound management [12].

Manuka honey has demonstrated relevant antimicrobial and immunomodulatory properties and is increasingly utilized in clinical wound care [13]. Rich in methylglyoxal (MGO), Manuka honey exhibits broad-spectrum bactericidal activity, particularly against antibiotic-resistant strains [14]. In addition, its acidic pH and high osmolarity facilitate autolytic debridement and promote granulation tissue formation [15,16]. Privrodski et al. formulated an alginate-based hydrogel incorporated with medical-grade Manuka honey and demonstrated superior healing in a porcine burn model: the treatment accelerated re-epithelialization, increased collagen deposition, and reduced macrophage activity compared to standard antibiotic ointments, underscoring the therapeutic value of Manuka honey–alginate composites [17]. Another widely studied molecule in tissue regeneration is hyaluronic acid (HA), a glycosaminoglycan naturally found in the extracellular matrix. Its ability to retain water, regulate cell signaling, and support keratinocyte and fibroblast proliferation makes it a valuable additive in hydrophilic biomaterials [18]. The viscoelastic and bioactive properties of HA contribute to faster re-epithelialization and reduced scar formation, particularly in the context of second- and third-degree burns [19].

Antioxidant protection also plays a critical role in wound healing by mitigating reactive oxygen species (ROS), which are elevated in burn wounds and associated with chronic inflammation and delayed tissue regeneration [20]. Plant-based extracts, such as those derived from *Ribes nigrum* (blackcurrant), are rich in anthocyanins and polyphenolic compounds with proven antioxidant and anti-inflammatory activity [21]. These bioactive agents can help regulate the oxidative environment of the wound bed, thus improving cellular viability and angiogenesis [22].

In the present work, we developed a multilayered, pH-responsive hydrogel dressing based on alginate enriched with Manuka honey, hyaluronic acid, and blackcurrant extract. Each layer was designed to address a specific therapeutic need: the Manuka honey layer for antimicrobial protection, the HA layer for hydration and cell support, and the blackcurrant extract layer for oxidative stress modulation. The system was fabricated using a dual-step ionic crosslinking strategy to ensure structural stratification and mechanical integrity while supporting the encapsulation of active compounds. The multilayer structure was coated with chitosan, a cationic polysaccharide with additional hemostatic, antiadhesive, and antibacterial properties, in order to improve pH-responsiveness [23]. The innovative design of this multilayer hydrogel aims to replicate the multifactorial therapeutic approach required for potential burn treatment, integrating antimicrobial protection, moisture retention, pH-responsiveness, and antioxidant activity in a single patch. A comprehensive physicochemical characterization was performed to assess the hydrogel’s functional performance. Fourier-transform infrared (FTIR) spectroscopy was employed to confirm the integration of bioactive compounds within the hydrogel matrix. Swelling behavior was evaluated under different pH conditions to investigate the responsiveness of each formulation in environments that mimic wound progression. Water vapor transmission rate (WVTR) was measured to evaluate the capacity of the material to regulate moisture exchange, a critical parameter for promoting re-epithelialization while preventing dehydration or maceration. Overall, the results highlight the potential of the multilayer hydrogel as a bioactive and responsive material for wound management.

## 2. Results and Discussion

### 2.1. Hydrogel Fabrication by Two-Step Ionic Crosslinking

**Figure 1:**
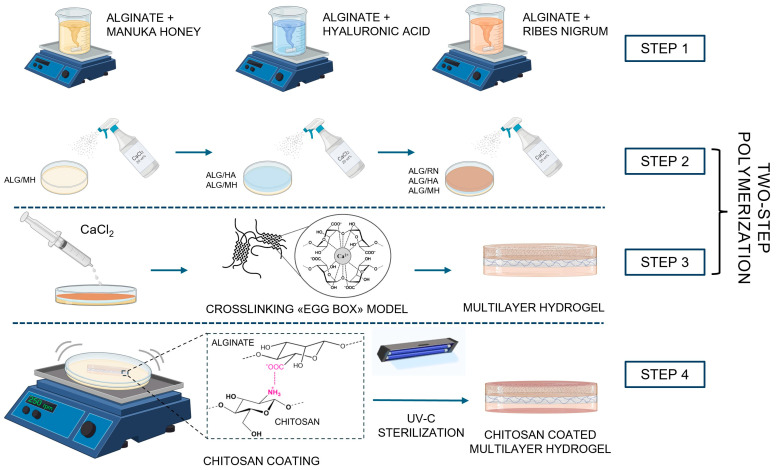
Schematic of the multilayer hydrogel fabrication process. The hydrogel was assembled by sequential casting of three layers. Manuka honey/alginate, hyaluronic acid/alginate, and blackcurrant extract/alginate crosslinked by dual-step ionic approach: surface gelation via CaCl_2_ spray (20 wt%) after each layer and final bulk crosslinking by immersion. The structure was subsequently coated with chitosan (0.5 wt%) and sterilized by UV-C and ozone treatment.

The multilayered hydrogel was developed through a sequential casting approach integrated with a dual-step physical crosslinking process, designed to ensure stratification, and interfacial stability without inducing interlayer diffusion. As schematically illustrated in Figure 1, three different biopolymer formulations were deposited in succession: alginate/Manuka honey (2 wt% Alg, 10 wt% MH), alginate/hyaluronic acid (2 wt% Alg, 0.3 wt% HA), and alginate/blackcurrant extract (2 wt% Alg, 1 wt% RN). These compositions were selected to confer complementary functionalities associated with wound care, including barrier protection, hydration, and antioxidant potential. The bottom layer (MH, 12 mL) was first cast into a Petri dish. Immediately after deposition, the surface was subjected to localized ionic crosslinking by nebulizing 2 mL of a 20 wt% calcium chloride (CaCl_2_) solution (~40 sprays). This step started physical gelation of the alginate matrix through divalent ion bridging of guluronic acid residues, forming the characteristic “egg-box” structures. The resulting surface-specific gelation was necessary to stabilize the interface, preventing interlayer mixing while maintaining sufficient internal hydration to ensure cohesion with subsequent layers. Following a 2 min stabilization period, the HA layer (9 mL) was cast on top and subjected to the same crosslinking process. Lastly, the RN layer (18 mL) was added, completing the stratified structure. To reinforce the overall network, a second global crosslinking step was performed by immersing the multilayered system in 10 mL of 20 wt% CaCl_2_ solution for 30 min, ensuring thorough ion diffusion and uniform network consolidation. A two-step crosslinking strategy, comprising a localized spray following each casting step and a final immersion, was systematically optimized to enhance interfacial definition, mechanical integrity, and network uniformity. The optimized protocol, involving the application of 2 mL of crosslinking solution, a 2 min contact period, and a subsequent 30 min immersion, consistently yielded reproducible and homogeneous structures. After crosslinking, the hydrogel was rinsed and immersed in a 0.5 wt% chitosan solution and subjected to vortex-based dynamic coating at 250 rpm for 30 min. Electrostatic complexation between chitosan amino groups and alginate carboxyl groups provided an additional layer of mechanical reinforcement and surface functionality. The final multilayered device was sterilized via combined UV-C (254 nm) and ozone (185 nm) exposure for 6 min. The resulting multilayered architecture was organized to achieve a hierarchical therapeutic distribution, with the bottom MH layer serving as a protective interface, the intermediate HA layer promoting water retention and network hydration, and the upper RN layer contributing to oxidative stress modulation.

The two-step ionic crosslinking strategy was selected because it provides mild gelation conditions, avoiding the use of organic solvents or photoinitiators and thus preserving the integrity of sensitive bioactive agents. In addition, this method ensures reproducible gel formation and facilitates the assembly of multilayer structures, which would be more challenging with covalent approaches. Nevertheless, ionically crosslinked alginate hydrogels are inherently limited by modest mechanical strength and gradual ion exchange with the surrounding medium, which can compromise long-term stability. In our system, the application of a chitosan coating was introduced to mitigate these drawbacks by enhancing barrier properties and structural robustness.

### 2.2. Swelling Properties and Morphological Characterization

**Figure 2:**
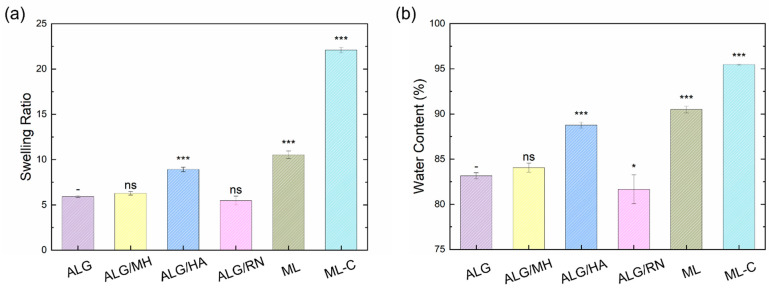
Swelling ratio (SR) (**a**) and water content (WC) (**b**) of alginate-based hydrogels, including single-layer formulations (ALG, ALG/MH, ALG/HA, ALG/RN), the multilayer (ML), and the chitosan-coated multilayer (ML-C). The data are presented as mean ± SD (*n* = 5). Statistical significance vs. ALG taken as the control (“-“) was assessed by one-way ANOVA followed by Dunnett’s post hoc test (ns *p* ≥ 0.05; * *p* < 0.05; *** *p* < 0.001).

Figure 2 shows the swelling ratio and water content (%) of uncoated hydrogels at room temperature (T = 25 °C), including pure alginate and the three functional layers (ALG/MH, ALG/HA, and ALG/RN) used in the multilayer patch, together with the multilayer (ML) and its chitosan-coated counterpart (ML-C). All samples exhibited high water content values (>80%), confirming the intrinsic hydrophilicity of alginate, which is rich in hydroxyl and carboxylic groups able to form hydrogen bonds with surrounding water molecules.

**Figure 3:**
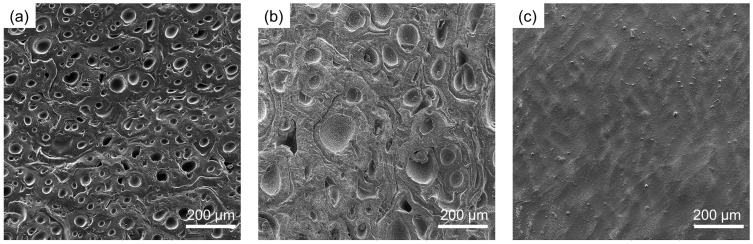
SEM images of dried hydrogel layers: (**a**) ALG/MH, (**b**) ALG/HA, and (**c**) ALG/RN.

Among the tested formulations, the alginate/hyaluronic acid (ALG/HA) hydrogel exhibited the highest swelling ratio (8.91) and water content (88.77%), highlighting the strong hydration capability of hyaluronic acid. This behavior is attributed to the presence of –OH, –COOH, and –NH groups in its structure, which contribute to extensive hydrogen bonding and enhanced the water uptake. The intermediate layer exhibits superior hydration properties, which may help maintain optimal moisture levels within the wound bed during healing. The ALG/MH formulation presented a moderate increase in water content (84.06%) and swelling ratio (6.28) compared to pure alginate (83.15% and 5.94, respectively). This can be explained by the hydrogen bonding interactions formed between the hydroxyl groups of alginate and the complex saccharidic structure of Manuka honey. In contrast, the ALG/RN formulation demonstrated the lowest hydration performance, with a swelling ratio of 5.48 and water content of 81.66%. This reduced water affinity may be due to the steric hindrance and lower polarity of some polyphenolic compounds present in the blackcurrant extract. To assess the performance of the multilayer configuration, swelling and water content tests were conducted on the multilayer hydrogel system, both with and without chitosan coating. As shown in Figure 2, the multilayer (ML) and the chitosan-coated multilayer (ML-C) showed higher SR and WC than the single-layer formulations. The uncoated multilayer hydrogel has a swelling ratio of 10.54 ± 0.42 and a water content of 90.50 ± 0.37%, outperforming the single-layer counterparts. This enhancement is likely the result of cumulative hydration from all constituent layers, especially the HA-rich intermediate layer, which acts as a water reservoir. Notably, the multilayer hydrogel coated with chitosan exhibited a significantly higher swelling ratio of 22.11 ± 0.25 and water content of 95.48 ± 0.05%. This marked increase reflects the high hydrophilicity of chitosan, which contains multiple –NH_2_ and –OH groups able to interact with water and alginate via electrostatic and hydrogen bonding. The coating not only reinforces the surface but also improves water uptake capacity. To further investigate the morphological features of the hydrogel layers, SEM imaging was performed on dried samples of ALG/MH, ALG/HA, and ALG/RN. Figure 3 shows the SEM images, highlighting clear differences among the three layers. The ALG/MH layer exhibited a porous, sponge-like structure with numerous cavities that appeared partially interconnected. The ALG/HA layer exhibited an intermediate morphology with larger and more rounded pores, consistent with its higher swelling ratio and water content, whereas the ALG/RN layer showed a compact and relatively smooth surface, with no evident porosity at the observed magnification. Surface porosity was quantified using ImageJ software (version 1.53) (*n* = 3 ROIs per sample). The analysis yielded porosity values of 45.1 ± 1.8% for ALG/MH and 30.5 ± 2.1% for ALG/HA. For ALG/RN, the automated image analysis indicated a minimal porosity fraction (<15%), likely attributable to image contrast and drying artifacts. These findings correlate with the hydration data, confirming that the higher swelling ratio and water content of ALG/HA are associated with a more open surface structure, while ALG/RN remains essentially non-porous.

### 2.3. Chemical Analysis by FTIR Spectroscopy

**Figure 4:**
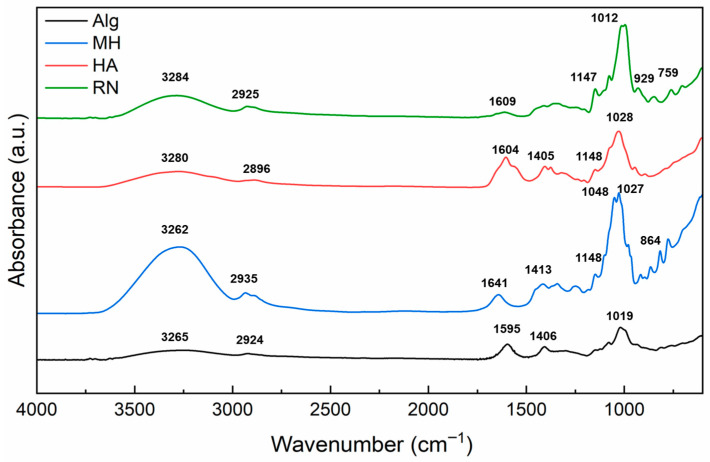
FTIR spectra of as-received compounds used in hydrogel formulation: sodium alginate (ALG), Manuka honey (MH), hyaluronic acid (HA), and blackcurrant extract (RN). The data are offset for clarity.

Figure 4 presents the FTIR spectra of the pristine compounds used in the fabrication of the hydrogel system: sodium alginate (ALG), Manuka honey (MH), hyaluronic acid (HA), and blackcurrant extract (RN). Spectra were recorded in the range of 4000–600 cm^−1^ to identify the characteristic vibrational modes associated with the functional groups present in each material.

The spectrum of alginate shows a broad absorption band in the region 3600–3000 cm^−1^, attributed to O–H stretching vibrations, characteristic of hydroxyl-rich polysaccharides [6]. A distinct peak at 2924 cm^−1^ corresponds to aliphatic C–H stretching [24]. The strong bands observed at 1595 cm^−1^ and 1406 cm^−1^ are ascribed to asymmetric and symmetric stretching of the carboxylate group (–COO^−^), respectively [5]. Peaks at 1019 cm^−1^ and 947 cm^−1^ reflect the C–O–C stretching of the polysaccharide backbone and the C–O vibration of uronic acid residues, confirming the typical fingerprint of alginate-based biopolymers [25].

In the FTIR spectrum of Manuka honey, a wide O–H stretching band was observed between 3380 and 3190 cm^−1^, along with C–H stretching vibrations in the 2850–3000 cm^−1^ region [24]. A distinct peak at 1641 cm^−1^ was attributed to C=O stretching vibrations from carbonyl-containing compounds, such as aldehydes and ketones present in saccharides [26]. Additional bands at 1413 cm^−1^ and in the 1027–1048 cm^−1^ range correspond to C–H deformation and C–O/C–C stretching, respectively, suggesting the presence of carbohydrates and polyols [27]. The spectrum of hyaluronic acid showed strong O–H and N–H stretching bands around 3280 cm^−1^, confirming its hydrophilic polysaccharide nature [28]. Peaks at 1604 cm^−1^ and 1405 cm^−1^ corresponded to asymmetric and symmetric –COO^−^ stretching, respectively [29]. A peak observed at 1374 cm^−1^ is attributed to –CH_2_ scissoring vibrations, whereas the bands at 1148 cm^−1^ and 1028 cm^−1^ are assigned to symmetric C–O–H bending and C–O stretching modes, respectively [29]. FTIR profiling of black currant extract revealed a broad -OH/N-H stretching band centered at 3284 cm^−1^ and a C-H stretching band at 2925 cm^−1^, both consistent with the characteristic vibrations observed in plant-derived polysaccharides [30,31]. The 1147 cm^−1^ band was assigned to asymmetric C–O–C stretching, commonly reported in glycosidic or ether linkages within pectic structures. The absorption band at 1352 cm^−1^ observed in the FTIR spectrum of the blackcurrant extract may be attributed to –CH_3_ bending or –CH_2_ scissoring vibrations, commonly found in plant-derived polysaccharides [30,31]. Although P=O stretching from phosphate-containing compounds is typically expected in the 1250–1150 cm^−1^ range [32], no clear signal in this region was observed. The signals at 1012 cm^−1^ and 995 cm^−1^ are consistent with pyranose ring vibrations, while the peaks at 929 cm^−1^ and 847 cm^−1^ correspond to α- and β-glycosidic bonds, confirming the presence of saccharide structures similar to those found in pectins and other plant polysaccharides [30,31].

**Figure 5:**
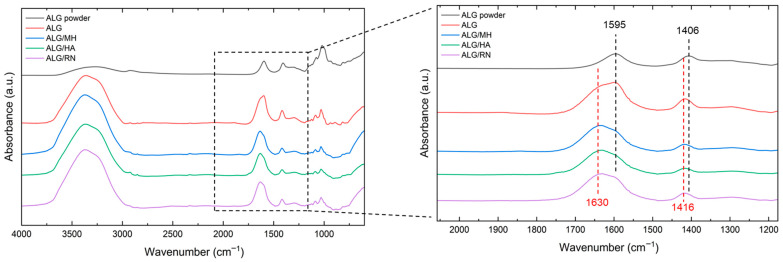
FTIR spectra of alginate powder, alginate hydrogel (ALG), and composite hydrogels incorporating Manuka honey (ALG/MH), hyaluronic acid (ALG/HA), and blackcurrant extract (ALG/RN). The enlarged view highlights the shifts in the carboxylate stretching bands following crosslinking and additive incorporation. The data are offset for clarity.

Figure 5 shows the FTIR spectra of the hydrogels synthesized with different bioactive compounds, Manuka honey (ALG/MH), hyaluronic acid (ALG/HA), and blackcurrant extract (ALG/RN), in comparison with pristine sodium alginate (ALG) and unprocessed alginate powder. All hydrogel spectra exhibit a broad absorption band in the 3600–3000 cm^−1^ region, corresponding to O–H stretching vibrations [6]. This feature, more pronounced than in the dry alginate powder, is indicative of the high water content typical of hydrated polymeric networks.

In the spectrum of pure alginate, a characteristic peak at 1595 cm^−1^ is associated with the asymmetric stretching of carboxylate groups (–COO^−^) [33]. Upon incorporation of the bioactive compounds, this peak undergoes a systematic red shift to ~1630 cm^−1^ across all three composite hydrogel formulations. This displacement is likely due to the presence of additional functional groups, possibly including conjugated or hydrogen-bonded C=O moieties from HA and MH. The shift suggests successful molecular entrapment within the alginate matrix and the occurrence of non-covalent interactions such as hydrogen bonding or electrostatic forces. Additionally, the peak at 1406 cm^−1^ observed in the dry alginate spectrum, attributed to symmetric carboxylate stretching, shifts to 1416 cm^−1^ in all crosslinked hydrogel samples [34,35]. This shift is ascribed to ionic crosslinking between the alginate matrix and divalent calcium ions (Ca^2+^) introduced during gelation. The coordination of Ca^2+^ with guluronic acid residues induces changes in electron density distribution around the carboxyl groups, resulting in the observed spectral shift. This phenomenon is consistent with the formation of “egg-box” structures typical of ionotropically crosslinked alginate hydrogels, as represented by the reaction [36]

2Na-Alg + CaCl_2_ → Ca-Alg + 2NaCl.

In the 1090–1030 cm^−1^ range, all hydrogel spectra displayed characteristic C–O–C and C–O stretching bands associated with glycosidic linkages. These signals, also present in the spectra of the individual components, confirm the retention of saccharide structures within the hydrogel network. The absence of new vibrational bands suggests that crosslinking and encapsulation occurred through physical interactions, without covalent modification.

### 2.4. Analysis of Hydrogel Water Vapor Transmission Rate (WVTR)

**Figure 6:**
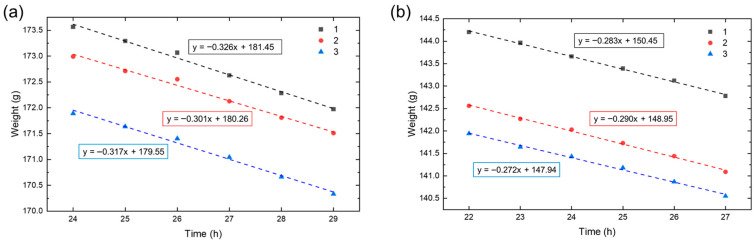
Linear regression of weight loss over time during WVTR testing for (**a**) uncoated and (**b**) chitosan-coated multilayer hydrogels. The tests were conducted in triplicates. The slope values represent steady-state evaporation rates used to calculate WVTR.

WVTR analysis was performed to assess the moisture management capabilities of the multilayer hydrogel under clinically relevant conditions. The test was conducted in a thermostatic chamber at 35 °C and low relative humidity (RH < 40%), in accordance with adapted ASTM E96 and EN ISO 7783:2019 standards [37,38]. Multilayer hydrogel patches, with and without chitosan coating, were compared to a negative control (PBS-filled container without any membrane) to quantify water vapor permeability. Figure 6 shows the time-dependent weight loss profiles for both uncoated and chitosan-coated multilayer hydrogels over a test duration of 29 and 27 h, respectively.

For uncoated samples, a steady-state transport regime, defined by a linear decrease in mass over at least six consecutive measurements, was reached after 24 h. In contrast, coated hydrogels exhibited linearity slightly earlier, at approximately 22 h. For each condition, the slope of the linear portion of the curve was used to calculate the WVTR, as described in Equation (3). To evaluate the contribution of hydrogel-derived water loss, blank samples without PBS were analyzed, and the corresponding weight change was subtracted from the total loss to isolate vapor transmission due solely to PBS evaporation. Table 1 reports the calculated WVTR values for the two multilayer configurations and the control. The chitosan-coated samples showed a reduced WVTR (3449.18 ± 87.51 g/m^2^·day) compared to uncoated hydrogels (3852.29 ± 128.42 g/m^2^·day), suggesting enhanced water retention capacity. A two-tailed Student’s t-test confirmed that this reduction was statistically significant (*p* < 0.01).

This outcome is consistent with the previously observed increase in swelling ratio and water content upon chitosan coating, attributed to its hydrophilic amine and hydroxyl groups. Although both values exceed the optimal WVTR (~2500 g/m^2^·day) generally recommended for burn wound dressings, they remain within the acceptable range for moist wound healing (up to 5000 g/m^2^·day) [39]. These results highlight the potential of the multilayered hydrogel system to manage moisture evaporation while preventing excessive accumulation of exudate.

### 2.5. pH-Responsive Swelling Kinetics

**Figure 7:**
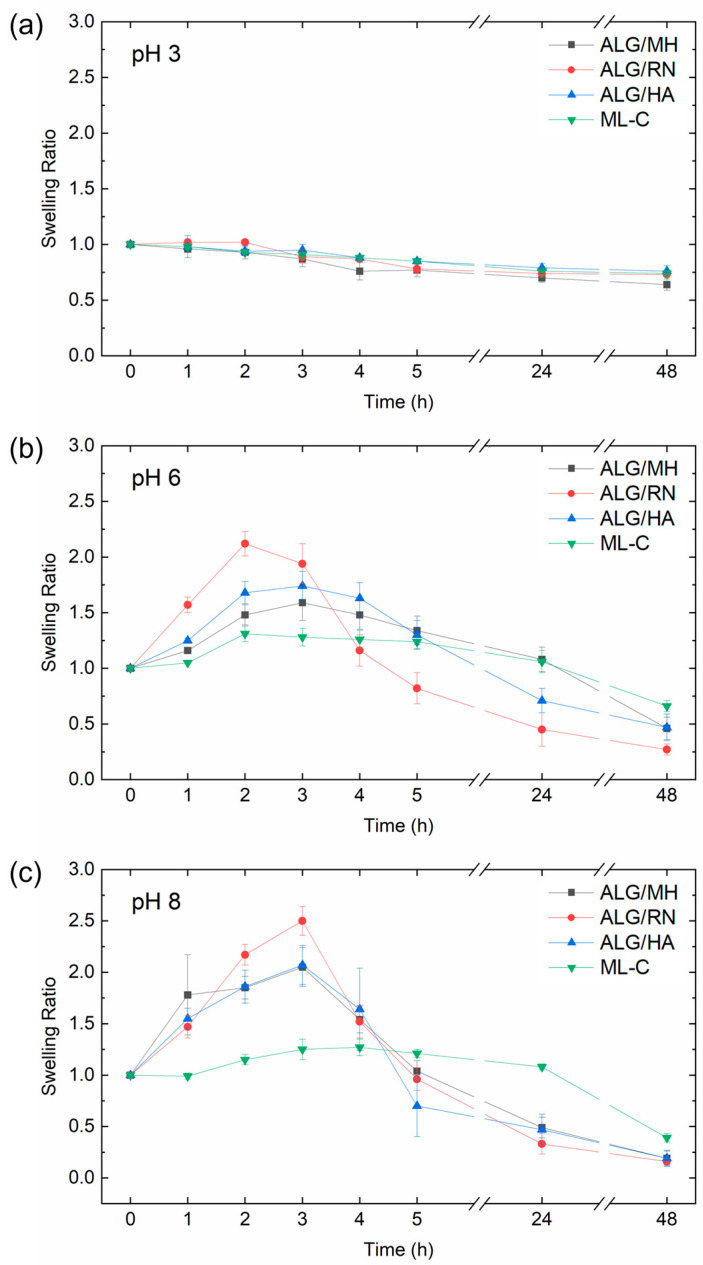
Swelling ratio of chitosan-coated hydrogel formulations (ALG/MH, ALG/RN, ALG/HA) and multilayer (ML-C) over 48 h under different pH conditions: (**a**) pH 3, (**b**) pH 6, and (**c**) pH 8 at 37 °C.

Figure 7 shows the swelling behavior of chitosan-coated hydrogels containing Manuka honey (ALG/MH), hyaluronic acid (ALG /HA), blackcurrant extract (ALG/RN) and the multilayer construct (ML-C) at three pH values (3, 6, and 8). The experiment was performed to evaluate the pH responsiveness of each formulation as an indicator of fluid uptake capacity under wound-like conditions. Hydrogels were incubated in phosphate-buffered saline (PBS) adjusted to pH 3, 6, and 8. These values simulate acidic healthy skin, alkaline inflamed wounds, and regenerating tissue, respectively. Swelling ratio (SR) was calculated over time using Equation (1). At pH 3, both alginate (pKa ≈ 3.6) and chitosan (pKa ≈ 6.5) remain predominantly protonated, resulting in low deprotonation of functional groups and a stable, compact hydrogel network. Consequently, all formulations displayed minimal swelling (SR ~0.6–0.7) with negligible changes over time. The limited dissociation prevents electrostatic repulsion, preserving the tight structure of the hydrogel matrix. At pH 6, alginate is nearly fully deprotonated (~99.6% COO^−^), while chitosan remains partially protonated (~24%), allowing partial electrostatic repulsion between polymer chains. This promotes a temporary loosening of the network and an increase in water uptake. All formulations showed a distinct swelling peak within the first 2–3 h (SR > 2.0), followed by a gradual decline, likely due to structural relaxation and beginning degradation.

At pH 8, both biopolymers are extensively deprotonated, with chitosan approaching 97% deprotonation. The complete loss of ionic interaction between -COO^−^ and -NH_3_^+^ groups leads to pronounced swelling (maximum SR > 2.5), especially in the ALG/RN system, followed by a more rapid structural collapse. The enhanced electrostatic repulsion between chains compromises the network integrity, accelerating water diffusion and potential material disintegration. Overall, these results demonstrate that all three hydrogel formulations exhibit a clear pH-responsive swelling profile, supporting their potential utility in dynamic wound environments where local pH can fluctuate significantly over time.

The multilayer hydrogel (ML-C) showed a comparable trend. The profile is similar to that observed in the single-layer systems. At pH 3, the swelling ratio remains stable over time, indicating a compact and ionically crosslinked network due to limited deprotonation of alginate and chitosan. At pH 6 and pH 8, the multilayer hydrogel shows an initial increase in swelling, followed by a gradual decrease. This reflects the pH-sensitive swelling–relaxation response seen in the individual layers, although the swelling peak appears later (after 4–5 h), suggesting slower water diffusion and relaxation, possibly due to interlayer interactions and additional crosslinking within the multilayer

### 2.6. In Vitro Degradation Tests

A degradation test was performed to evaluate the stability of the hydrogels under different pH conditions (3, 6, and 8), simulating wound environments at various healing stages. The test followed the same setup as the swelling study. Hydrogel disks (*n* = 3) of each formulation, alginate/Manuka honey (ALG/MH), alginate/hyaluronic acid (ALG/HA), alginate/blackcurrant extract (ALG/RN), and the multilayer construct (ML-C), were hydrated and incubated in PBS at 37 °C for 48 h. After incubation, samples were weighed, and the percentage weight loss was calculated using Equation (4). Results are summarized in Table 2.

All single-layer hydrogels exhibited low degradation at pH 3 (weight loss ~24–36%), consistent with the protonated state of both alginate and chitosan, which promotes ionic crosslinking and structural stability. At pH 6, degradation increased markedly, with weight losses reaching ~73% for ALG/RN, due to alginate deprotonation (COO^−^ ~99.6%) and weakened interaction with partially deprotonated chitosan (NH_3_^+^ ~24%). At pH 8, both polymers were almost completely deprotonated (COO^−^ ~100%, NH_2_ ~97%), and ionic crosslinks were lost. This resulted in extensive degradation (>80% mass loss) in all monolayers. In contrast, the multilayer hydrogel showed improved stability at all pH values. At pH 3, its behavior was similar to the monolayers (−26.4%), but at pH 6 and 8, degradation remained limited (−33.7% and −41.4%, respectively). This suggests that multilayer assembly, combined with interfacial chitosan coating and dual-step crosslinking, enhances structural cohesion and slows degradation.

**Figure 8:**
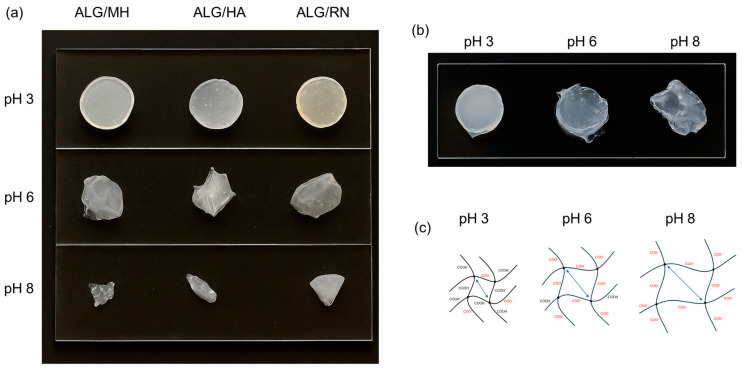
Degradation behavior of hydrogel systems after 48 h at different pH values: (**a**) single-layer hydrogels and (**b**) multilayer hydrogel. (**c**) Schematic representation of pH-induced deprotonation and resulting electrostatic repulsion between negatively charged chains, promoting hydrogel degradation.

Figure 8 shows the visual outcomes of the degradation test under different pH conditions. Single-layer hydrogels remained intact at pH 3 but exhibited visible disintegration at pH 6 and 8 (Figure 8a). In contrast, the multilayer system retained its structure even in alkaline media, confirming improved stability (Figure 8b). Figure 8c presents a schematic illustration of pH-dependent network evolution, where progressive deprotonation disrupts ionic crosslinks and promotes matrix loosening.

To assess the potential pH-modulating activity of the hydrogel formulations, the pH of each PBS solution was measured before and after the in vitro degradation test. As shown in Table 3, the pH remained stable under acidic conditions, while a marked decrease was observed in initially neutral and alkaline PBS, particularly with the multilayer hydrogel. At an initial pH of 8, the final pH dropped to 5.10 in the presence of the multilayer, suggesting acidification of the medium. This behavior is likely due to the release of acidic functional groups or buffering actions of the incorporated bioactive components.

These results underline the added functionality of the multilayer hydrogel, which not only resists degradation in alkaline conditions but also contributes to restoring an acidic environment. This property is particularly relevant for burn wound care, where a sustained acidic pH is associated with antimicrobial protection and improved healing. The observed pH modulation, along with the hydrogel’s stability and swelling behavior, highlights its potential as a responsive dressing for dynamic wound environments.

### 2.7. Kinetic Release Studies of Bioactive Principles

**Figure 9:**
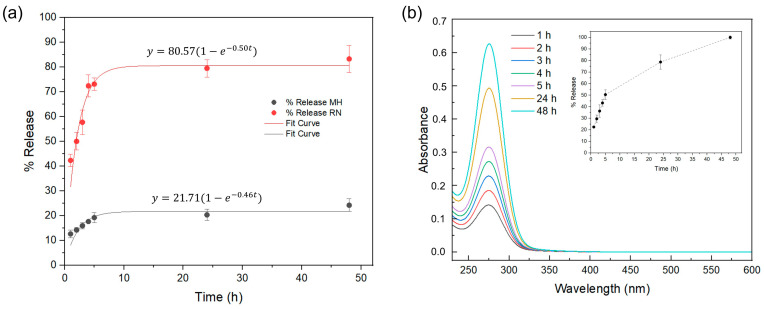
Release profiles of bioactive principles from alginate hydrogels determined by UV-vis spectroscopy. (**a**) Release (%) of Manuka honey (MH) and blackcurrant extract (RN) from single-layer hydrogels, fitted with a first-order kinetic model. (**b**) Representative UV–vis spectra for the multilayer system at selected time points, with inset showing the relative release normalized to the absorbance at 48 h.

UV–vis spectroscopy was used to monitor the release of Manuka honey (MH) and blackcurrant extract (RN) from the alginate hydrogels in time (Figure 9) under wound environment conditions (PBS at pH 8.0, 37 °C). For the single-layer systems, concentrations at each time point were determined using calibration curves and the release percentage calculated with respect to the total content of the bioactive principle in the gel.

Figure 9a shows that both ALG/MH and ALG/RN follow apparent first-order release kinetics. The blackcurrant-containing hydrogel (ALG/RN) shows a faster release (k ≈ 0.50 h^−1^), reaching an asymptote around 80% after the first 6 h, in agreement with the high diffusivity of small hydrophilic phenolics through hydrated alginate networks [40]. In contrast, ALG/MH released more slowly (k ≈ 0.46 h^−1^, plateau ≈ 20–25%), consistent with the stronger hydrogen bonding and viscosity of the sugar-rich Manuka phase, which is known to hinder diffusion and interact with alginate chains [41].

The multilayer formulation showed an intermediate profile of the release kinetics (Figure 9b), reflecting the superposition of a rapid RN-driven burst and a slower MH-governed contribution. Here, independent calibration was not feasible due to overlapping absorption maxima of MH and RB extract at 275–276 nm, and the absorbance was therefore normalized to the value at 48 h and treated as a relative cumulative signal (inset in Figure 9b).

## 3. Conclusions

In this study, a bioinspired multilayer hydrogel patch was successfully fabricated using an optimized dual-step ionic crosslinking strategy. The multilayer system was designed to organize active compounds into spatially distinct layers, each contributing with specific physicochemical properties relevant to wound care. The bottom layer, based on Manuka honey, was designed to be in contact with the wound, since antibacterial properties and acidity may aid in local infection control and pH modulation. The middle layer, containing hyaluronic acid, was intended to support hydration, while the upper layer, enriched with blackcurrant (*Ribes nigrum*) extract, was formulated to contribute with antioxidant activity. Alginate served as the structural matrix for all layers, chosen for its biocompatibility and high-water content, mimicking the hydration of soft tissue. FTIR spectroscopy confirmed the successful incorporation of all active compounds into the polymeric network. The chitosan-coated multilayer hydrogel exhibited superior water uptake (SR = 22.11 ± 0.25) and high-water content (95.48 ± 0.05%) compared to single-layer systems. The water vapor transmission rate (WVTR) further supports the patch’s ability to maintain a moist environment favorable to wound healing, while allowing suitable gas exchange. The system also demonstrated pH-responsive behavior, with controlled swelling in alkaline conditions, suggesting adaptability to dynamic wound environments. Degradation tests confirmed its enhanced structural stability across pH variations, with weight loss below 42% at pH 8, in contrast to degradation observed in single-layers, which is higher than 80%. Furthermore, the multilayer patch induced a pH reduction under alkaline conditions, highlighting a potential buffering effect that may help restore physiological acidity in chronic wounds. Release studies of the bioactive principles under wound environmental conditions further supported the layer-specific functions observed in the physicochemical assays: the blackcurrant layer released a higher fraction of its load than the layer with Manuka honey, while the multilayer exhibited an intermediate profile consistent with sequential release. Overall, these results highlight the functional advantages of the multilayer configuration and the effectiveness of the fabrication strategy. The proposed patch represents a promising system for structurally stable, moisture-retentive, and pH-responsive hydrogel dressings suitable for potential burn wound management. Further studies are required to validate the bioactivity of the multilayer hydrogel system and to confirm the specific functions of each individual layer. In addition, future work will focus on the in vivo validation of the multilayer hydrogel using an animal wound or burn model to evaluate its therapeutic efficacy and clinical applicability.

## 4. Materials and Methods

Alginic acid sodium salt, calcium chloride (CaCl_2_) dihydrate and chitosan (medium molecular weight, >75% deacetylated) were purchased from Sigma-Aldrich (Milan, Italy). Hyaluronic acid sodium salt (purity ≥ 95%, molecular weight 1500–1800 kDa) and black currant leaf dry extract (*Ribes nigrum* L.) (extraction solvent: water; drug-to-extract ratio: 4:1; particle size ≥ 90% through 300 µm; loss on drying ≤ 5%) were purchased from ACEF S.p.A. (Fiorenzuola d’Arda, Italy). Manuka honey (MGO 515+, UMF 15+) (methylglyoxal content ≥ 514 mg/kg) was purchased from Steens (Masterton, New Zealand). Deionized water (resistivity 18.2 MΩ⋅cm) was produced by a Direct-Q3 UV water purification system (Millipore, Molsheim, France) and used in all preparations.

### 4.1. Fabrication of Multilayered Hydrogel

The multilayered hydrogel system was developed through a sequential casting strategy employing three distinct formulations: alginate/Manuka honey (ALG/MH), alginate/hyaluronic acid (ALG/HA), and alginate/blackcurrant extract (ALG/RN). Each solution was prepared in ultrapure water under controlled mixing and temperature conditions to ensure complete polymer dissolution and formulation homogeneity. A 2 wt% sodium alginate stock solution was first prepared by dissolving the polymer in ultrapure water. This solution served as the reference matrix for all subsequent formulations. For the RN formulation, 1 wt% of blackcurrant (*Ribes nigrum*) leaf dry extract was incorporated into the alginate matrix. The suspension was continuously stirred for 60 min to promote complete dispersion and solubilization of the botanical compound. In the HA formulation, 0.3 wt% sodium hyaluronate was added to the alginate solution and stirred at 50 °C for 1 h. This thermal-assisted protocol facilitated the full hydration of the high-molecular-weight glycosaminoglycan, yielding a clear solution. To obtain the MH formulation, Manuka honey (MGO 515+, UMF 15+; methylglyoxal content ≥ 514 mg/kg) was initially solubilized in ultrapure water at concentrations of 5 wt%, 10 wt%, or 15 wt%. The mixture was stirred at 30 °C for 10 min to preserve the structural integrity and bioactive properties of the honey. Following this step, sodium alginate was added to achieve a final concentration of 2 wt%, and the system was stirred at room temperature (T = 25 °C) for an additional 45 min to ensure uniform gel precursor formation. For the ionic crosslinking steps, a 20 wt% calcium chloride (CaCl_2_·2H_2_O) solution was prepared by dissolving the salt in ultrapure water with continuous stirring for 30 min until full dissolution. Lastly, a 0.5 wt% chitosan solution was obtained by chitosan (degree of deacetylation >75%) in a 5% v/v aqueous acetic acid solution (5 mL acetic acid in 95 mL water). The mixture was magnetically stirred for 1 h.

### 4.2. Morphological and Swelling Analyses

The morphology of the multilayered hydrogel was examined using a Dino-Lite AM7915MZT digital microscope (Dino-Lite Europe IDCP B.V., Almere, The Netherlands) equipped with DinoCapture 2.0 software.

Swelling behavior was assessed by immersing each hydrogel layer (MH, HA, RN) in deionized water until equilibrium was reached, followed by drying at 50 °C for 18 h. The swelling ratio and water content were determined using Equations (1) and (2), respectively [7,42,43]:

(1) $$Swelling\;Ratio=\frac{W_{s}}{W_{d}},$$

(2) $$Water\;Content(\%)=\frac{\left(W_{s}-W_{d}\right)}{W_{s}}\times 100,$$

where W_s_ is the weight of the hydrogel in the equilibrium swelling state, and W_d_ is the weight of the fully dried hydrogel. The test was repeated on five samples for each hydrogel layer (MH, HA, RN), as well as for the complete multilayer, both with and without chitosan coating, and the results averaged.

Statistical significance was evaluated by one-way analysis of variance (ANOVA) followed by Dunnett’s post hoc test, with ALG taken as the control. Values of *p* < 0.05 were considered significant. Significance levels are indicated as ns (*p* ≥ 0.05), * (*p* < 0.05), ** (*p* < 0.01), and *** (*p* < 0.001).

### 4.3. Morphological Characterization

The morphology and microstructure of the hydrogel layers were evaluated by scanning electron microscopy (SEM) using a VEGA II LSH instrument (Tescan, Brno, Czech Republic). Samples were dried at 50 °C for 18 h, sputter-coated with gold using a Cressington 108 auto sputter coater (Cressington Scientific Instruments, Watford, UK), and imaged at an accelerating voltage of 10 kV with magnifications of 300×. Surface porosity was quantified by 2D image analysis of the SEM images using ImageJ software (version 1.53) developed by the National Institute of Health (NIH, Bethesda, MD, USA).

### 4.4. FTIR Analysis

Fourier transform infrared (FTIR) spectra were recorded using a Nicolet™ Summit/Everest™ spectrometer (Thermo Fisher Scientific, Waltham, MA, USA) equipped with a zinc selenide attenuated total reflectance (ATR) accessory. Spectra were acquired over the range 4000–600 cm^−1^ with a resolution of 4 cm^−1^, and each spectrum was obtained by averaging 64 scans. Background interference from ambient air was subtracted prior to analysis. Initially, the neat compounds (sodium alginate, Manuka honey, sodium hyaluronate, and blackcurrant extract) were characterized to identify their individual spectral fingerprints. Subsequently, ATR-FTIR analyses were conducted on the hydrogel layers (MH, HA, RN) to evaluate compositional features and intermolecular interactions.

### 4.5. Water Vapor Transmission Rate (WVTR)

The water vapor transmission rate (WVTR) of the multilayer hydrogel patches was determined using a gravimetric method adapted from ASTM E96 [37] and EN ISO 7783:2019 [38], to evaluate their ability to regulate vapor exchange, a key parameter in the design of wound dressings, particularly for burn care. The multilayered hydrogel patch, with and without the chitosan coating, was mounted over the open mouth of a cylindrical container filled with 120 mL of phosphate-buffered saline (PBS). The distance between the liquid surface and the patch was fixed at 2 cm. A silicone gasket, a porous support mesh, and a locking ring were used to hermetically seal the sample, ensuring that water vapor could pass exclusively through the hydrogel membrane. The sealed assemblies were then placed in a closed thermostatic chamber maintained at 35 °C with (relative humidity < 40%), corresponding to the average skin temperature in burn conditions [44]. A blank control was prepared using an identical setup but without any membrane, to monitor direct PBS evaporation and exclude the contribution from the hydrogel. All setups were weighed at 1 h intervals until a steady-state regime was achieved, defined by a linear and constant weight decrease over at least six consecutive measurements, with negligible water loss in the control. The WVTR was calculated by applying linear regression to the mass loss over time during the steady-state phase, using the following equation [45]

(3) $$WVTR=\frac{G/t}{A},$$

where G/t is the slope of the weight loss curve (g/h) and A is the exposed area of the hydrogel patch (m^2^).

This protocol enabled a quantitative comparison of water vapor permeability between coated and uncoated multilayered hydrogels, providing critical insight into their potential performance as wound dressing materials. The analysis was carried out on three independent replicates (*n* = 3) for both the uncoated and chitosan-coated multilayer hydrogels, enabling a comparative evaluation of their vapor permeability. A two-tailed Student’s t-test was performed to assess the statistical significance of the difference between coated and uncoated multilayers.

### 4.6. Swelling Kinetics Under Simulated Wound pH Conditions

To evaluate the pH-dependent swelling kinetics of the hydrogel system under physiologically relevant conditions, tests were performed in phosphate-buffered saline (PBS) at pH 3, 6, and 8. These values were selected to mimic the wound microenvironment throughout the burn healing process [46]. Intact skin typically maintains an acidic pH (4.7–5.75), which becomes increasingly alkaline post-burn (up to pH 9.5, mean ≈ 8.1), before gradually returning to acidic conditions during tissue repair. Hydrogel disks (1.5 cm in diameter, 1–3 mm thick) from each layer (MH, HA, RN) and from the complete multilayer (*n* = 3 per group) were coated with chitosan using vortex (30 min at 250 rpm) and weighed in their initial hydrated state. The samples were then immersed in PBS at pH 3, 6, or 8 and incubated at 37 °C. At predefined time points (1, 2, 3, 4, 5, 24, and 48 h), samples were retrieved, gently blotted to remove surface liquid, and reweighed. The swelling ratio (SR) was calculated according to Equation (1), enabling a quantitative assessment of the temporal evolution of hydrogel dimensional changes under variable pH conditions.

### 4.7. Dissolution Behavior and pH-Responsive Stability

The dissolution profile of the hydrogel samples was investigated under the same simulated physiological conditions used in the swelling experiments. Triplicate specimens (*n* = 3) from each hydrogel formulation—alginate/Manuka honey (ALG/MH), alginate/hyaluronic acid (ALG/HA), alginate/blackcurrant extract (ALG/RN), and the complete multilayer assembly—were prepared as circular disks (1.5 cm diameter, 1–3 mm thickness), hydrated, and then immersed in 15 mL of PBS at pH 3, 6, and 8. All samples were incubated at 37 °C to mimic physiological temperature. After 48 h, the disks were removed, lightly blotted, and weighed. The percentage weight loss was calculated using the following equation

(4) $$Weight\;Loss(\%)=\frac{W_{i}-W_{f}}{W_{i}}\times 100,$$

where W_i_ and W_f_ represent the initial and final hydrated weights of the hydrogel specimens, respectively. To understand the phenomenon of degradation as pH changes, the deprotonation rate of carboxyl groups was calculated using the Henderson–Hasselbalch equation [47]:

(5) $$Deprotonation(\%)=\frac{10^{\left(pH-pKa\right)}}{10^{\left(pH-pKa\right)}+1}\times 100,$$

where pK_a_ is the acid dissociation constant of the carboxylic group involved. This formula states the relationship between the pH of the environment and the percentage of deprotonated carboxylic groups at that specific pH value.

### 4.8. In Vitro Release Studies

In vitro release of Manuka honey and *Ribes nigrum* compounds from the hydrogels was quantified by UV–Vis spectroscopy (UV-2700, Shimadzu, Kyoto, Japan) using 10 mm quartz cuvettes and neat PBS solution for the baseline. Cylindrical hydrogel disks (area 1 cm^2^; three disks per time point) were each immersed in 10 mL of PBS solution with pH 8.0 and at 37 °C to mimic the alkaline environment of burn wounds. The release medium was collected at predefined time intervals (1, 2, 3, 4, 5, 24, and 48 h) and analyzed. UV-vis spectra were acquired in the 200–1200 nm range and processed in the 230–600 nm window encompassing the absorption maxima (λ_max_) of the bioactive principles. For single-layer formulations (ALG/RN and ALG/MH), concentrations were determined using calibration curves in PBS (blackcurrant, 5–45 mg L^−1^; Manuka honey, 100–2000 mg L^−1^) after linear regression of the absorbance data. The release (%) was expressed as percentage of the total mass of the compound present in the hydrogel before incubation. For the multilayer hydrogel (ML), the characteristic λ_max_ values of Manuka honey (276 nm) and blackcurrant extract (275 nm) overlapped, resulting in a single cumulative peak at 275 nm. In this case, no independent calibration curves could be established, and the cumulative release was estimated directly from the absorbance signal, normalized as [48]

(6) $$\%\;Relative\;Release\left(t\right)=\frac{A_{t}}{A_{48h}}\times 100,$$

where A_t_ is the absorbance at time *t* and A_48h_ the value at 48 h.

Normalized release profiles were fitted using a first-order kinetic model:

(7) $$y=A(1-e^{-kt})$$

where A is the maximum theoretical release (%), k the release rate constant, and t the incubation time (h).

## Figures and Tables

**Table 1:** Water vapor transmission rate (WVTR) values of multilayer hydrogel dressings with and without chitosan coating, compared to PBS-only negative control. Values represent mean ± standard deviation (*n* = 3).

Sample Type	WVTR (g/m^2^·Day)
Multilayer hydrogel (uncoated)	3852.29 ± 128.42
Multilayer hydrogel (chitosan-coated)	3449.18 ± 87.51
Negative control (PBS only)	5681.63 ± 10.00

**Table 2:** Weight loss (%) of chitosan-coated single-layer and multilayer (ML-C) hydrogel formulations after 48 h incubation at pH 3, 6, and 8 at 37 °C. Values are presented as mean ± SD (*n* = 3).

Sample		Weight Loss (%)	
	pH 3	pH 6	pH 8
ALG/RN	−26.6 ± 1.2	−73.2 ± 5.2	−83.9 ± 3.1
ALG/HA	−24.1 ± 0.8	−52.9 ± 11.7	−80.6 ± 7.8
ALG/MH	−36.0 ± 4.7	−54.2 ± 10.4	−80.9 ± 6.5
ML-C	−26.4 ± 7.1	−33.7 ± 5.1	−41.4 ± 3.8

**Table 3:** pH values of PBS solutions after 48 h of incubation with different hydrogel samples.

Sample	pH 3	pH 6	pH 8
ALG/MH	2.60 ± 0.04	4.35 ± 0.13	6.92 ± 0.14
ALG/HA	2.60 ± 0.02	4.20 ± 0.05	6.55 ± 0.11
ALG/RN	2.61 ± 0.04	4.33 ± 0.10	6.90 ± 0.11
ML-C	2.67 ± 0.02	3.92 ± 0.01	5.10 ± 0.06

## Data Availability

The data are available from the corresponding author upon reasonable request.
